# Supplementary material for: Saturated and Polyunsaturated Fatty Acids Production by Aurantiochytrium limacinum PKU#Mn4 on Enteromorpha Hydrolysate
Source: Mar Drugs. 2023 Mar 23;21(4):198. doi: 10.3390/md21040198 (PMC10143273; doi:10.3390/md21040198)
Supplement: Supplementary file 1 [file marinedrugs-21-00198-s001.zip › marinedrugs-2257084-supplementary.pdf]

Supplementary Information

Saturated and Polyunsaturated Fatty Acids Production by *Aurantiochytrium* sp. PKU#Mn4 on *Enteromorpha* Hydrolysate

Yaodong He[1,2†], Xingyu Zhu[1,†],Yaodong Ning[1], Xiaohong Chen[1], Biswarup Sen[1]*, Guangyi Wang[1,3,4]*

[1]Center of Marine Environmental Ecology, School of Environmental Science and Engineering, Tianjin University, Tianjin 300072, China

[2]School of Fishery, Zhejiang Ocean University, Zhoushan 316022, China

[3]Key Laboratory of Systems Bioengineering (Ministry of Education), Tianjin University, Tianjin 300072, China

[4]Qingdao Institute for Ocean Technology of Tianjin University Co., Ltd., Qingdao 266237, China

†These authors contributed equally

*Corresponding author

Email: bsen@tju.edu.cn

gywang@tju.edu.cn

Table S1. Composition of *Enteromorpha* hydrolysate medium.

Enteromorpha (g)	Sterile distilled water (mL)	98% (w/v) H_2SO_4 (mL)
40	760	8
60	1140	12
80	1520	16
100	1900	20
120	2280	24

Table S2. Fatty acid composition of lipids accumulated during the cultivation of PKU#Mn4 on different concentrations of *Enteromorpha* hydrolysate.

**Fatty acid**	***Enteromorpha* hydrolysate (g/L)**				
	**40**	**60**	**80**	**100**	**120**
C12:0	0.43 ± 0.01	0.59 ± 0.02	0.54 ± 0.03	0.45 ± 0.03	0.59 ± 0.01
C14:0	2.03 ± 0.07	2.15 ± 0.21	2.83 ± 0.08	2.95 ± 0.11	3.44 ± 0.17
C15:0	5.22 ± 0.34	3.69 ± 0.25	8.13 ± 0.39	5.29 ± 0.19	8.87 ± 0.27
C16:0	31.22 ± 1.54	31.06 ± 1.87	32 ± 2.33	34.25 ± 1.65	33.73 ± 0.77
C17:0	1.11 ± 0.02	1.31 ± 0.22	2.1 ± 0.13	1.47 ± 0.1	1.91 ± 0.09
C18:0	2.67 ± 0.22	3.36 ± 0.2	2.13 ± 0.33	2.21 ± 0.02	1.67 ± 0.06
C20:4n-6	0.55 ± 0.01	0.69 ± 0.02	0.68 ± 0.03	0.63 ± 0.01	0.75 ± 0.02
C20:5n-3	2.77 ± 0.03	2.82 ± 0.05	3.16 ± 0.06	2.61 ± 0.11	3.22 ± 0.09
C22:5n-3	7.56 ± 0.72	8.82 ± 0.17	7.95 ± 0.33	8.48 ± 0.56	7.37 ± 0.44
C22:6n-3	37.22 ± 2.44	39.13 ± 1.78	35.99 ± 1.25	37.4 ± 2.1	35.59 ± 1.23
SFA	42.65 ± 3.2	42.18 ± 2.67	47.76 ± 3.31	46.64 ± 2.9	49.64 ± 1.89
PUFA	48.12 ± 1.69	51.48 ± 2.78	47.79 ± 2.55	49.13 ± 1.08	46.95 ± 1.17

Data are expressed as mean ± SD of triplicate experiments. The values (mean ± SD) represent the percent of TFA. SFA and PUFA are acronyms for saturated fatty acids and polyunsaturated fatty acids, respectively. Lipids were extracted at the end of the fermentation.

Table S3. Fatty acid composition of lipids accumulated during the cultivation of PKU#Mn4 strain in different concentrations of glucose.

**Fatty acid**	**Glucose (g/L)**				
	**10**	**20**	**30**	**40**	**50**
C12:0	0.34 ± 0.02	0.12 ± 0.01	0.28 ± 0.01	0.51 ± 0.02	0.21 ± 0.02
C14:0	5.87 ± 0.16	4.37 ± 0.22	5.54 ± 0.91	7.25 ± 0.65	3.38 ± 0.05
C15:0	0.99 ± 0.65	1.77 ± 0.1	1.67 ± 0.01	0.63 ± 0.04	0.59 ± 0.03
C16:0	36.19 ± 3.71	40.66 ± 2.63	41.12 ± 3.06	40.33 ± 2.92	31.88 ± 2.83
C17:0	0.02 ± 0	0.05 ± 0	0.11 ± 0.01	0.03 ± 0	0.01 ± 0
C18:0	0.63 ± 0.08	0.79 ± 0.06	1.35 ± 0.08	0.97 ± 0.03	1.03 ± 0.01
C20:4n-6	0.24 ± 0.02	0.44 ± 0.01	0.12 ± 0.01	0.1 ± 0	0.28 ± 0.02
C20:5n-3	0.47 ± 0.01	0.32 ± 0.02	0.25 ± 0.01	0.49 ± 0.02	0.39 ± 0.02
C22:5n-3	7.74 ± 0.04	6.79 ± 0.12	6.23 ± 0.09	4.22 ± 0.13	4.48 ± 0.24
C22:6n-3	43.26 ± 3.78	41.98 ± 3.22	37.6 ± 4.73	39.05 ± 3.97	40.11 ± 4.61
SFA	44.09 ± 3.67	47.73 ± 2.79	50.07 ± 4.55	49.72 ± 2.92	37.15 ± 3.37
PUFA	51.64 ± 2.49	49.5 ± 3.65	44.23 ± 2.44	43.84 ± 1.73	45.22 ± 1.46

Data are expressed as mean ± SD of triplicate experiments. The values represent the percent of total fatty acids. SFA and PUFA are acronyms for saturated fatty acids and polyunsaturated fatty acids, respectively. Lipids were extracted at the end of the fermentation.

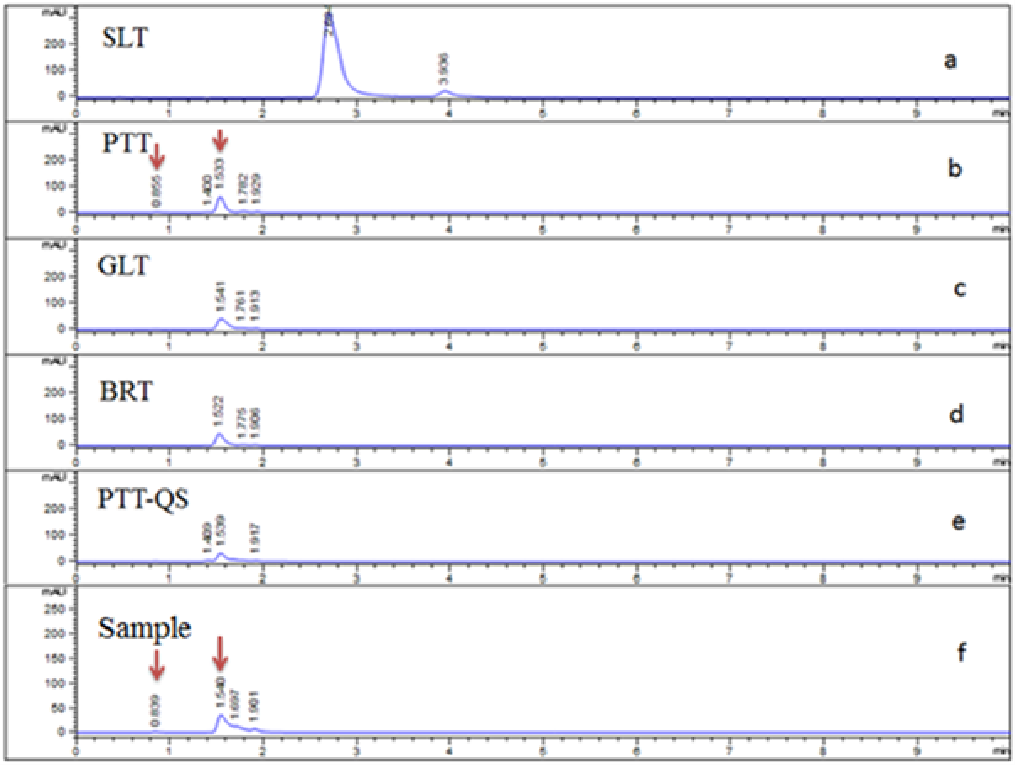

Figure S1. HPLC analysis of monosaccharide standard solution. (a) Rhamnose, (b) Glucose, (c) Mannose, (d) Galactose, (e) Glucuronic acid, and (f) *Enteromorpha* hydrolysate.
